# Proteomic analysis of the chemosensitizing effect of curcumin on CRC cells treated with 5-FU

**DOI:** 10.3389/fmed.2022.1032256

**Published:** 2022-11-24

**Authors:** Jingbo Yang, Chengyan He, Ning Liu

**Affiliations:** ^1^Central Laboratory, Second Hospital, Jilin University, Changchun, China; ^2^Clinical Laboratory, China-Japan Union Hospital, Jilin University, Changchun, China

**Keywords:** proteomics, curcumin, CRC, 5-FU, ROS

## Abstract

**Background:**

5-Fluorouracil (5-FU) is one of the most common chemotherapy drugs used to treat colorectal cancer (CRC), which often develops resistance in more than 15% of patients. Curcumin, an active component of Curcuma longa, has been reported to show antitumor activity in CRC and, furthermore, enhance the effect of chemotherapy against colorectal cancer cells. However, the molecular mechanisms underlying the sensitizing effect of curcumin on 5-FU have not been largely elucidated. In this study, we aimed to systematically investigate the role of curcumin as a chemosensitizer for the treatment of CRC, along with the key events responsible for its pharmaceutical effect, which may lead to better clinical outcomes.

**Methods:**

A high-resolution 2DE-based proteomics approach was used to characterize global protein expression patterns in CRC cells treated with 5-FU both in combination with curcumin or without. The differentially expressed proteins were obtained from the 2DE analysis and subsequently identified by MALDI-TOF MS or nano-ESI-MS/MS, some of which were validated by the Western blot. Intracellular reactive oxygen species (ROS) were measured to assess the change in the redox environment resulting from the drug treatment.

**Results:**

A series of proteins with altered abundances were detected and identified by MALDI-TOF or nano-MS/MS. From a total of 512 isolated proteins, 22 proteins were found to be upregulated and 6 proteins were downregulated. Intracellular ROS was significantly elevated after curcumin treatment. Furthermore, mass spectrometry data revealed that some of the proteins appeared to have more oxidized forms upon curcumin treatment, suggesting a direct role for ROS in the chemosensitizing effect of curcumin.

**Conclusion:**

The effect of curcumin in enhancing chemosensitivity to 5-FU is a complex phenomenon made up of several mechanisms, including enhancement of the intracellular level of ROS. Our findings presented here could provide clues for a further study aimed at elucidating the mechanisms underlying the chemosensitizing effect of curcumin.

## Introduction

Colorectal cancer is a common malignant tumor of the digestive tract with a complicated and multifaceted parthenogenesis (1). It has been well-recognized that both genetic factors and living environment can induce the occurrence of colorectal cancer. Nowadays, the incidence and mortality of colorectal cancer have shown a rapid increase all over the world (2). In China, with the changes in the living environment and dietary habits, the mortality rate of colorectal cancer is also rising rapidly. The incidence rate of colorectal cancer in China has become equal to the world average (3, 4). Currently, methods for the treatment of colorectal cancer include radical surgery, postoperative radiotherapy, and chemotherapy (5–7). Early colorectal cancer can be treated with radical surgery, while in advanced metastatic colorectal cancer, the opportunity for surgery is lost and only chemoradiotherapy and other treatment means can be used though the prognosis is often poor with a 5-year survival rate of only 11%. At present, the clinical treatment of early colorectal cancer is mainly surgical radical resection, followed by 5-fluorouracil (5-FU) combined with other chemotherapy drugs (oxaliplatin, irinotecan, etc.) as postoperative adjuvant chemotherapy, which can further improve the disease-free survival (DFS) and/or overall survival (OS) of patients (8–10). Unfortunately, multiple chemotherapies often lead to drug resistance, which is also a major obstacle affecting the efficacy and prognosis of chemotherapy (11, 12). Curcumin, an active component extracted from Curcuma longa, has been shown to affect the sensitivity of tumor cells to chemotherapeutic drugs, including 5-FU (13). However, the molecular mechanisms underlying the sensitizing effect of curcumin on chemotherapeutic drugs have not been largely explored, which will eventually contribute to the establishment of new treatment strategies to improve drug efficacy, which is of great significance to improving clinical efficacy.

Curcumin and its anti-tumor effects has been subject to extensive exploitation as a third-generation cancer chemopreventive drug for several malignant tumors such as gallbladder cancer, liver cancer, and gastrointestinal cancer (14–16). Although curcumin as an anti-cancer agent has entered the stage of clinical trials, the outcome from some of the clinical trials was not very satisfactory for a variety of reasons (17). Some clinical trials failed because of the low concentration of curcumin adopted in the treatment, while others using high doses of curcumin displayed serious toxic reactions due to its genotoxicity and long-term effects (18). Therefore, it is very urgent for researchers to systematically investigate the detailed mechanisms underlying the pharmaceutical potentials of curcumin as an efficient anti-cancer agent in clinical applications.

Since the concept of the proteome was first proposed in 1994, the field of proteomics has been developing rapidly, providing a high-throughput technological platform for in-depth and systematic research on various life phenomena and their mechanisms, as well as the pathogenesis of various major human diseases, from a dynamic, multidisciplinary, and holistic perspective (19–21). At present, extensive proteomic investigations have been carried out in multiple human tumor tissues or cell lines, including colorectal cancer (CRC), whereas limited proteomic studies have focused on the anti-tumor effect of curcumin on CRC. For example, Lee et al. compared the proteomes of primary and metastatic colorectal cancer cell lines, SW480 and SW620, respectively, which were treated with different chemotherapy agents and natural compounds. The results showed that oxaliplatin, ginsenoside 20(S)-Rg3, and curcumin displayed significant anti-tumor activity, which mainly affected fatty acid synthase and histone H4 (22). In another example using an analog of curcumin with an alkyne moiety that can be conjugated with functional moieties through click chemistry, a list of proteins in HCT116 cells that were bound to curcumin were identified, suggesting that curcumin may target EIF2, eIF4/p70S6K, and mTOR signaling pathways. In addition, mitochondrial dysfunction could be induced by curcumin (23). Although some achievements have been made by several proteomics studies on the anti-cancer potential of curcumin, the underlying multifaceted mechanisms remain unclear and need to be further explored.

In the present study, we adopted 2DE coupled with mass spectrometry to systematically identify the key proteins, as well as the key events, involved in the chemosensitizing effect of curcumin on the CRC cells treated with 5-fluorouracil (5-FU), aiming to further decipher the underlying molecular mechanisms that may eventually lead to better clinical outcomes.

## Methods

### Cell culture

Human CRC SW480 cells were cultured in DMEM supplemented with 1% penicillin-streptomycin and 10% fetal bovine serum (FBS) at 37°C in 10-cm dishes under a humidified 5% CO_2_ atmosphere. Curcumin, as well as 5-fluorouracil, dissolved in DMSO was added into the culture media at different final concentrations.

### Cytotoxicity assay

The cytotoxic activities of the two compounds, namely, curcumin and 5-fluorouracil, toward SW480 cells were measured using the MTT assay. Briefly, 1 × 10^4^ cells per well were seeded into 96-well culture plates and cultured for 48 h at 37°C. Then, the culture media were replaced with fresh DMEM containing curcumin and/or 5-fluorouracil and incubated for an additional 48 h. The culture media were then replaced by freshly prepared media containing 0.5 mg/ml MTT. After 4 h of incubation, the resulting insoluble purple formazan was dissolved with 200 μl DMSO. A microplate reader was used to measure the absorbance at 570 nm to calculate the cytotoxicity of the drugs. Triplicate measurements were performed for each concentration of the drugs.

### Protein sample preparation

The cells that were used for proteomics analyses were washed with a Tris-buffered 250 mM sucrose solution and collected using a cell scraper. Then, the cells were lysed with a freshly prepared lysis buffer (8 M urea, 4% w/v CHAPS, and 50 mM DTT), which was supplemented with a cocktail of protease inhibitors. The protein samples were obtained from cell lysates by ultracentrifugation at 16,000 × *g* at 4°C for 30 min. The samples were subjected to a DC-RC protein assay and then stored at −80°C until used for 2DE.

### 2DE

The protein samples that were subjected to 2DE were prepared by dilution of 0.5 mg protein into 300 μl with rehydration solution, followed by loading into IPG strips (24 cm, pH 3–10 nonlinear, Amersham) for 12 h. Isoelectric focusing was carried out for a total of 70,000 V-h. Then, the IPG strips were equilibrated, and the proteins in the IPG strips were separated by the second-dimensional SDS electrophoreses. After 2DE separation, the gels were detached from glass plates and fixed immediately in 10% TCA for 60 min. The proteins in the gels were stained with Coomassie Brilliant Blue G-250. The gel images were scanned with a scanner. The PDQuest software was used to analyze the gel images, by which the total density on each gel was normalized to accurately compare spot quantity between gels.

### In-gel digestion and MS analysis

The protein spots of interest were destained and then subjected to in-gel digestion by TPCK-trypsin for 12 h at 37°C. The tryptic peptides were purified by ZipTip C18 tips before the MS analysis. Most of the tryptic peptide samples were analyzed using a Voyager DE STR MALDI TOF mass spectrometer (Applied Biosystems). A saturated CHCA solution was used as a matrix, which was mixed with peptide samples and then loaded on the sample plate. Besides MALDI TOF MS, nano-ESI-MS/MS was performed on some of the tryptic peptide samples by using a QSTAR mass spectrometer (Applied Biosystems). The tryptic peptide sample was loaded onto a PicoTip emitter and then ionized through an external nanoelectrospray ion source. The ions with multiple charge states were manually selected for MS/MS analysis to obtain the data for their fragment ions. Both MS and MS/MS data were searched against the human subset in the SwissProt database using the MASCOT software to identify the protein spots.

### Western blot

For validation of the results from proteomics analyses, the cell lysates obtained in urea/thiourea lysis buffer (refer to the “Cytotoxicity assay” section) were mixed 1:1 with denaturing loading buffer and subjected to SDS-PAGE 12%. The separated proteins were then transferred onto PVDF membranes. Proteins of interest on the membranes were probed using primary antibodies such as anti-PRDX6 polyclonal antibody (Invitrogen), anti-PDI monoclonal antibody (Santa Cruz Biotechnology), and anti-dUTPase monoclonal antibody (Santa Cruz Biotechnology), followed by incubation with properly diluted secondary antibodies conjugated with horseradish peroxidase. The signals of each protein were then visualized using an ECL reagent.

### ROS assay

A DCFH-DA probe was used to detect intracellular ROS levels. The DCFH-DA probe can be captured by cells and enter the cell. After cell metabolism, it is oxidized by intracellular ROS to generate fluorescent products, which can be detected by FCM (flow cytometry). SW480 cells were seeded in six-well plates for 24 h, then curcumin and 5-FU were added to each well at different concentrations (0, 20, 50, and 100 μM), and the culture was continued for 48 h. Triplicate the cells for each concentration of the drug. Then, the cells were collected and washed three times with PBS. The DCFH-DA probe was added to the cell suspension and incubated for 20 min in the dark. The cells were washed three times with PBS, resuspended with PBS, and the fluorescence signal intensity was assayed using FCM (flow cytometry).

## Results and discussion

### Curcumin can increase the cytotoxicity of 5-FU on SW480 cells

**Figure 1:**
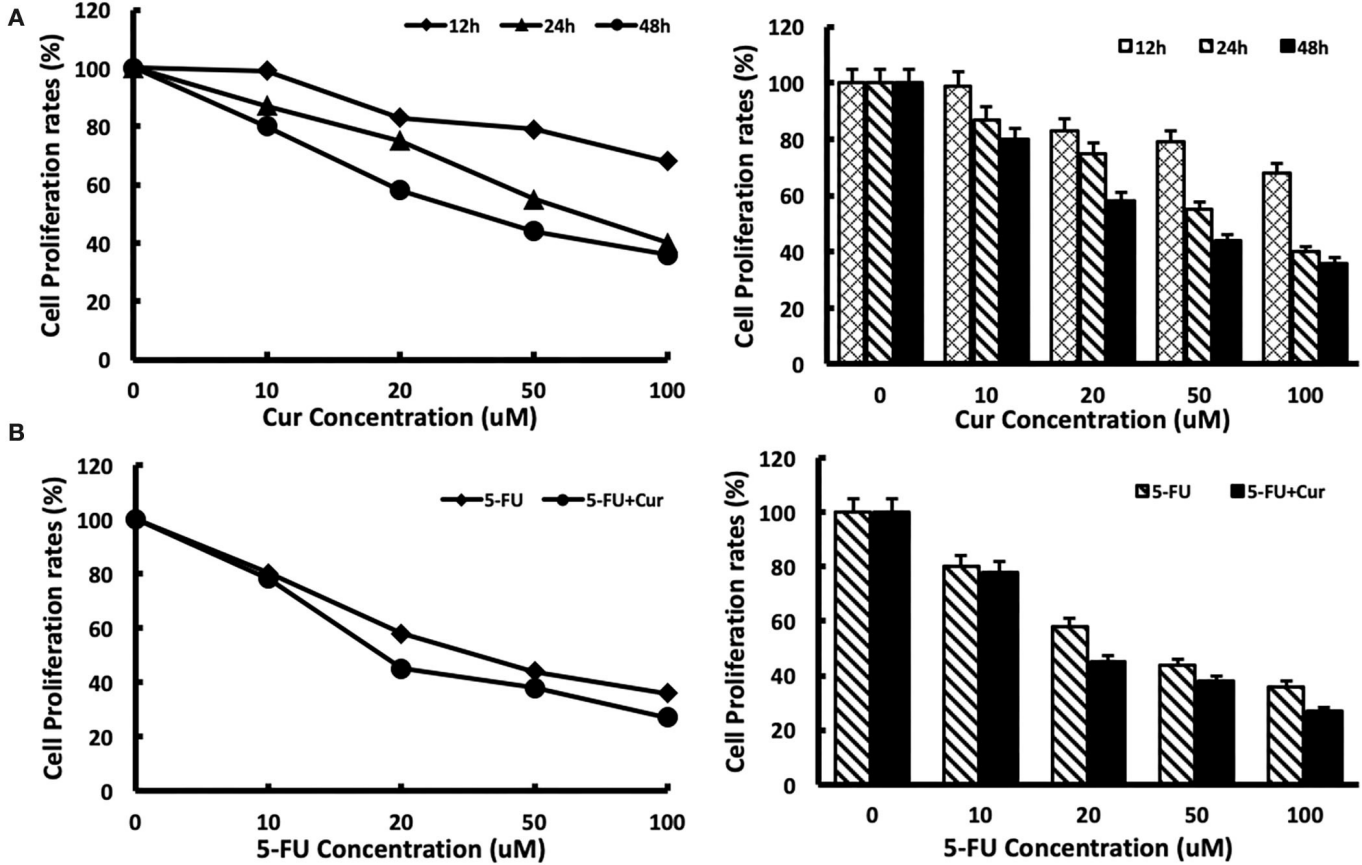
Cytotoxicity of 5-FU in SW480 cells is enhanced by curcumin. **(A)** Cytotoxicity of curcumin was determined after exposure of SW480 cells to curcumin with different concentrations (0, 10, 20, 50, and 100 μM) and different times (12 h, 24 h, and 48 h). Cell viability was measured with the MTT analysis. **(B)** Cytotoxicity of 5-FU was determined after exposure of SW480 cells to 5-FU alone and 5-FU in combination with 5 μM curcumin for 48 h. Cell viability was measured with the MTT analysis. The results are provided as mean values with standard deviations from at least three independent experiments.

To measure the inhibitory effect of curcumin on the viability of SW480 cells, we used MTT analysis to test the viability of SW480 cells treated with different concentrations (0, 10, 20, 50, and 100 μM) of curcumin for different incubation times (12, 24, and 48 h). The results showed that with the increase in curcumin concentration, the growth inhibition rate of SW480 cells decreased significantly. The inhibitory effect of curcumin was maximal after 48 h of incubation. The IC50 of curcumin was 30 μM at 48 h. No inhibition of the viability of SW480 cells was observed at the concentration of 5 μM. The results are shown in Figure 1A. Then, we tested the inhibitory effect of different concentrations (0, 10, 20, 50, and 100 μM) of 5-FU on the viability of SW480 cells with or without low concentration (5 μM) curcumin for 48 h. The results showed that, compared with those treated with 5-FU alone, the viability of SW480 cells decreased significantly with the increase of 5-FU concentration in a dose-dependent way: in the cells treated with 5-FU alone, when the concentration of 5-FU was 10 μM, the growth inhibition rate was significantly inhibited. When the concentration of 5-FU was 100 μM, the growth inhibition rate was at its maximum, and the IC50 value was about 40 μM. In the cells treated with both 5-FU and 5 μM curcumin, the growth inhibition rate was significantly enhanced compared with those treated with the same concentration of 5-FU, and the IC50 value was reduced to 20 μM. The results are shown in Figure 1B.

### Proteomics analysis identified relevant proteins targeted by curcumin treatment

**Figure 2:**
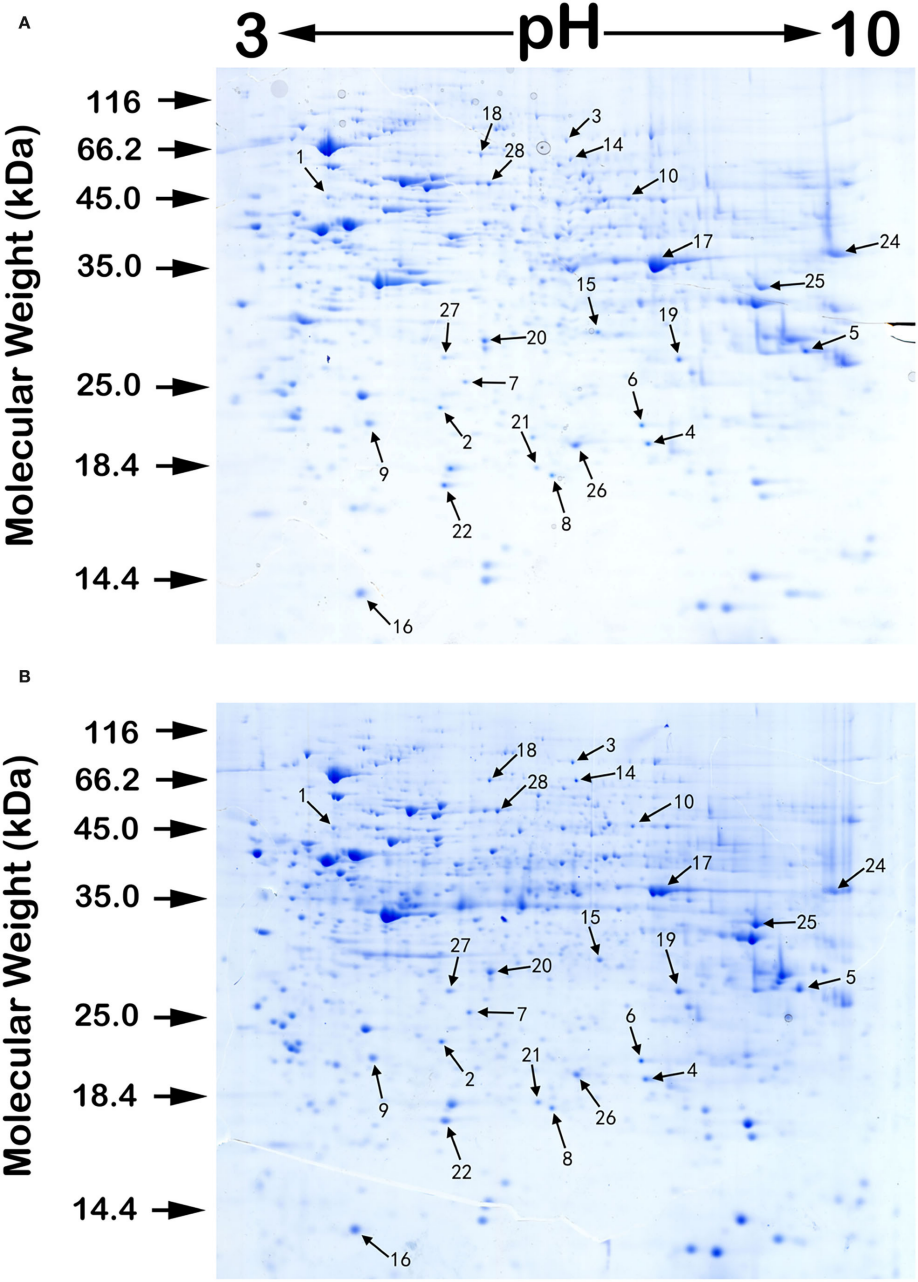
A pair of protein 2DE images of SW480 cells treated with 5-FU alone **(A)** and those treated with both 5-FU and curcumin **(B)** with sample loading of 0.5 mg protein each. The isoelectric focusing was carried out on 24 cm IPG strips with a nonlinear pH range of 3–10. Then, the proteins in the IPG strips were separated by the second-dimensional SDS electrophoreses. The gels were stained by Colloidal Coomassie blue G-250. Numbers associated with the spots on the gel images refer to the identified proteins listed in Table 1.

**Figure 3:**
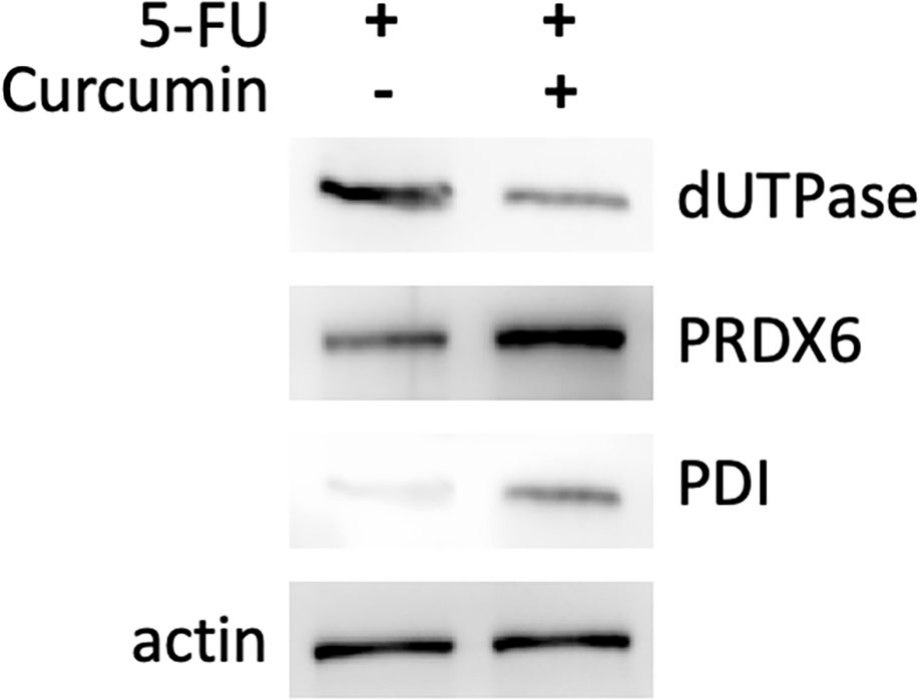
Western blotting analysis of three differentially expressed proteins: PRDX6, PDI, and dUTPase. Differential expression of the protein of interest between SW480 cells treated with 5-FU (20 μM) in combination with or without curcumin is normalized to actin level.

We performed proteomic analysis on the SW480 cells treated with 20 μM 5-FU alone and those treated with 20 μM 5-FU and 5 μM curcumin. Total proteins were extracted from the collected cells and then separated by high-resolution 2DE (Figure 2). The PDQuest software was used to compare the protein spot patterns of the gel images. A total of 28 differentially expressed protein spots between the two experimental groups were detected to be responsible for curcumin treatment. Among these proteins, 22 proteins were upregulated and 6 proteins were downregulated upon treatment with a combination of 5-FU and curcumin. These protein spots were subjected to MALDI-TOF-MS or nano-ESI-MS/MS analyses and subsequently identified by database searching (Table 1). From the identified protein candidates, peroxiredoxin-6 (PRDX6), protein disulfide-isomerase (PDI), and deoxyuridine 5'-triphosphate nucleotidohydrolase (dUTPase) were validated by Western blot analysis, and the expression changes were consistent with the 2DE results as shown in Figure 3.

**Table 1:** Summary of differentially expressed proteins (DEP) in SW480 cells in response to 5-FU and curcumin treatment.

**Spot number**	**Accession number**	**Protein information**	**Gene name**	**Observed pI/Mw (kDa)**	**Sequence coverage%**	**Theoretical pI/Mw (kDa)**
**Upregulated proteins in response to curcumin treatment**						
1	P27797	Calreticulin	CALR	4.02/47.4	28	4.29/48.14
2	Q04917	14-3-3 protein eta	YWHAH	4.90/25.3	24	4.76/28.2
3	P10809	60 kDa heat shock protein, mitochondrial	HSPD1	5.25/55.4	31	5.7/61.02
4	P49720	Proteasome subunit beta type-3	PSMB3	6.45/22.3		6.13/22.95
5	P15559	NAD(P)H dehydrogenase [quinone] 1	NQO1	8.45/35.5	24	8.91/30.87
6	P30041	Peroxiredoxin-6	PRDX6	5.45/23.0	19	6.0/25.03
7	P25788	Proteasome subunit alpha type-3	PSMA3	5.35/25.5	27	5.19/28.43
8	Q14152	Eukaryotic translation initiation factor 3 subunit A	EIF3A	5.33/24.0	27	6.38/116.57
9	P52565	Rho GDP-dissociation inhibitor 1	ARHGDIA	5.24/22.2		5.01/23.21
10	Q5EBM0	UMP-CMP kinase 2, mitochondrial	CMPK2	6.42/49.8	35	6.57/49.45
14	P17987	T-complex protein 1 subunit alpha	TCP1	5.88/61.5	22	5.8/60.34
15	Q15366	Poly(rC)-binding protein 2	PCBP2	6.41/42.4		6.33/38.58
16	P05386	60S acidic ribosomal protein P1	RPLP1	4.35/12.4		4.21/11.51
17	P06733	Enolase 1	ENO1	6.92/47.7	34	7.01/47.17
18	P07237	Protein disulfide-isomerase	P4HB	4.90/59.3	39	4.76/57.12
19	P32322	Pyrroline-5-carboxylate reductase 1, mitochondrial	PYCR1	7.01/30.7	35	7.18/33.36
20	P09972	Aldolase C, fructose-bisphosphate	ALDOC	6.10/40.9	26	6.14/39.46
21	P15531	Nucleoside diphosphate kinase A	NME1	6.27/20.0	31	5.81/17.15
22	P60660	Myosin light polypeptide 6	MYL6	5.11/18.1	26	4.56/16.93
**Downregulated proteins in response to curcumin treatment**						
24	P39023	60S ribosomal protein L3	RPL3	10.00/42.8	26	10.19/46.11
25	P22626	Heterogeneous nuclear ribonucleoproteins A2/B1	HNRNPA2B1	9.02/39.3	32	8.97/37.43
26	P33316	Deoxyuridine 5′-triphosphate nucleotidohydrolase, mitochondrial	DUT	5.95/20.5	38	6.15/17.75
27	P12429	Annexin A3	ANXA3	5.89/35.8	24	5.62/36.38
28	P52597	Heterogeneous nuclear ribonucleoprotein F	HNRNPF	5.08/47.0	33	5.37/45.67

**Figure 4:**
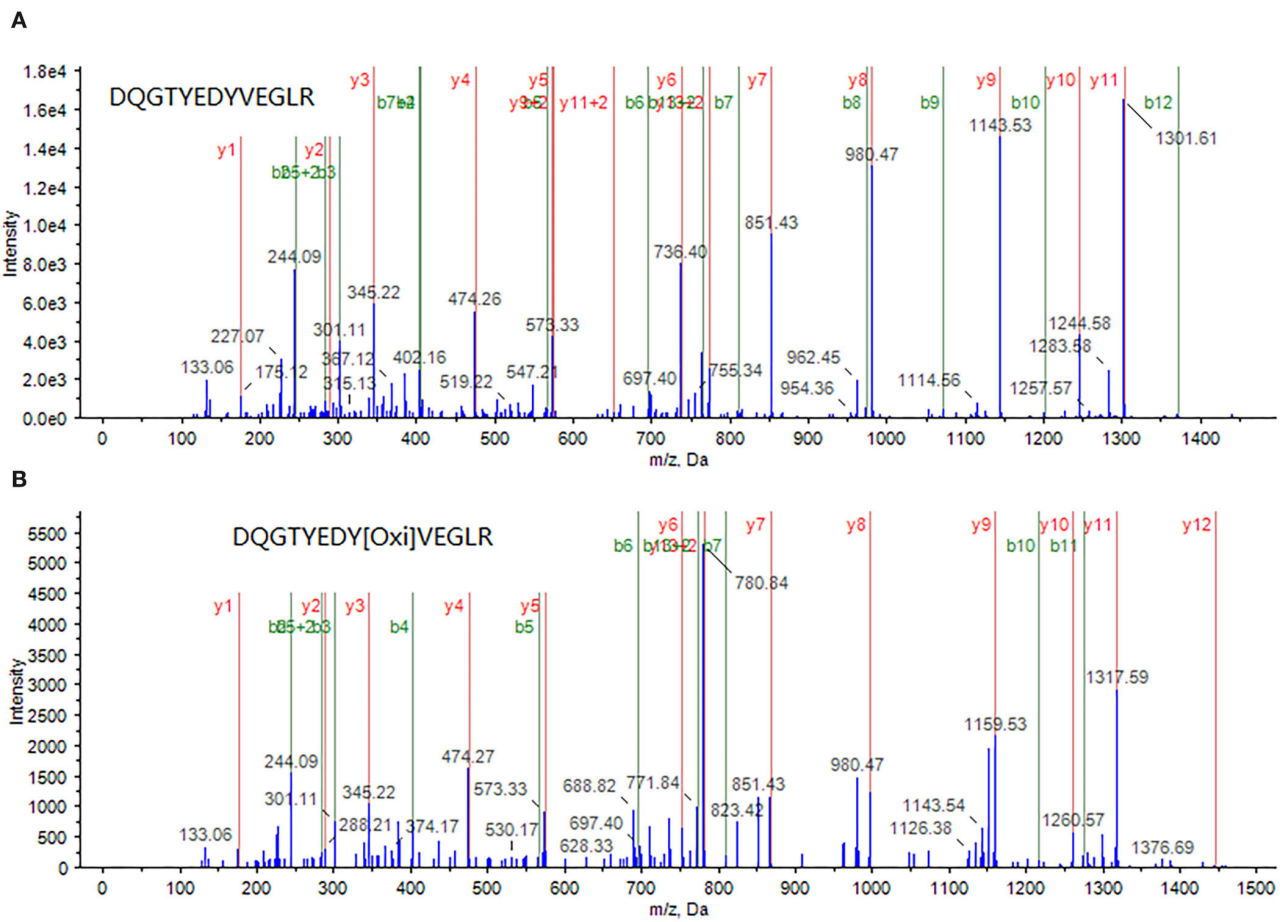
A peptide DQGTYEDYVEGLR (82–94) from myosin light polypeptide 6 was identified through MS/MS spectrum of a doubly-charged peak at m/z 772.8 **(A)**, whereas its oxidized form, in which the tyrosine (Y) is oxidized, was identified through MS/MS spectrum of a doubly charged peak at m/z 780.7 **(B)**.

Through an in-depth examination of the mass spectra of some differentially expressed proteins, evidence of the oxidized form of some peptides was discovered in cells treated with a combination of curcumin and 5-FU, implying an elevated oxidative environment in these cells. For example, in the samples treated with a combination of curcumin and 5-FU, myosin light polypeptide 6 appeared to be oxidized at a tyrosine residue within its peptide DQGTYEDYVEGLR (82–94), as shown in Figure 4.

### Intracellular ROS level was significantly increased upon curcumin treatment

**Figure 5:**
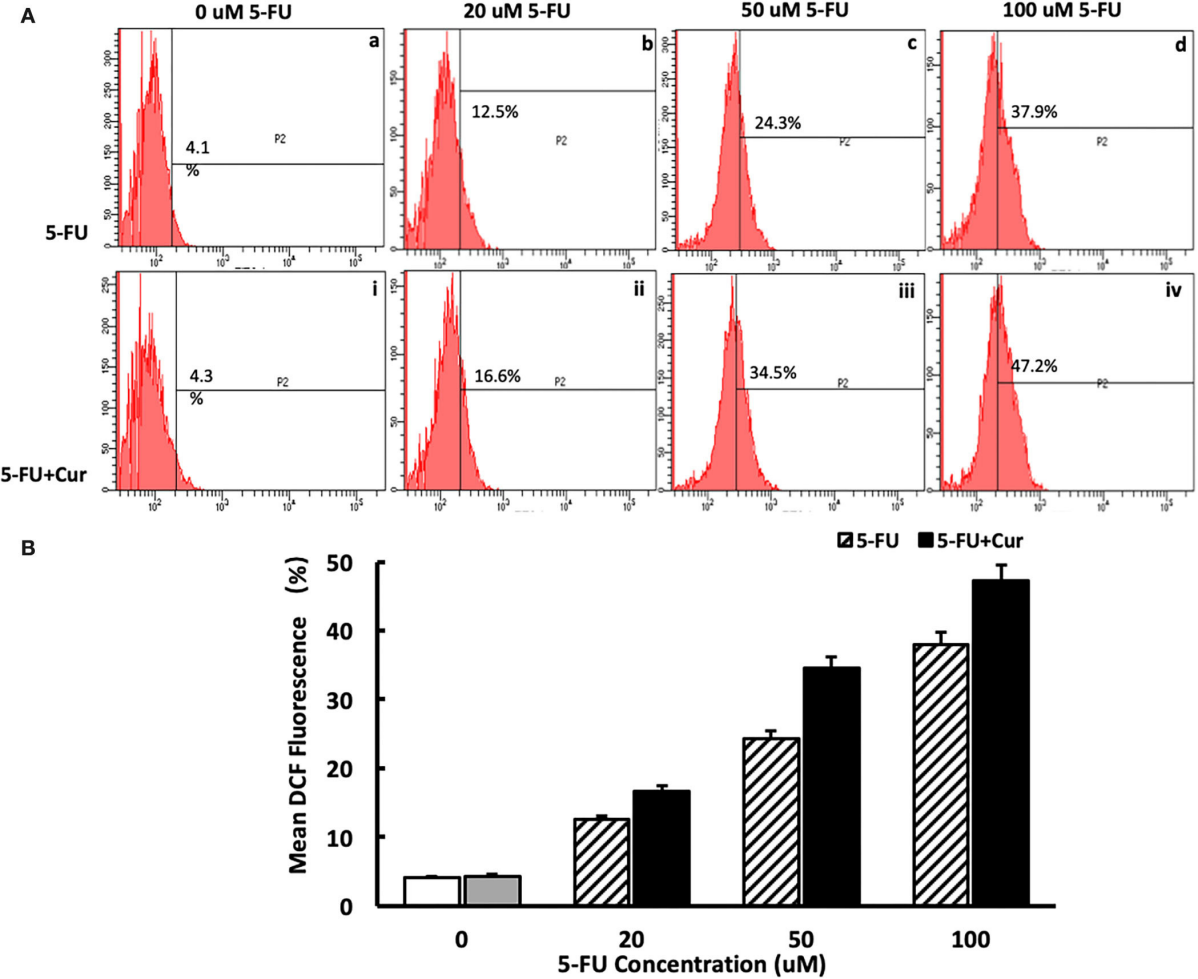
Curcumin can increase the ROS level in SW480 cells treated with 5-FU. [**(A)**: a–d] ROS level in SW480 cells treated with 5-FU (0, 20, 50, and 100) alone was slightly higher than that of the cells untreated, in a dose-dependent way. [**(A)**: i–iv, **(B)**] ROS level in SW480 cells treated with 5-FU (0, 20, 50, and 100 μM) and curcumin increased significantly compared to those in the cells treated with 5-FU alone in a dose-dependent manner.

In the process of apoptosis mediated by the mitochondrial pathway, with the increase of the degree of mitochondrial membrane potential depolarization, the occurrence of an oxidative stress response will be activated, leading to an increase in ROS level and promotion of the process of apoptosis. Therefore, ROS levels are an important marker of mitochondrial pathway-mediated apoptosis. In this study, ROS levels were measured in SW480 cells treated with different concentrations of 5-FU (0, 20, 50, and 100 μM). For the cells treated with 5-FU alone, the ROS level of SW480 cells was slightly higher than that of the cells untreated, in a dose-dependent way, as shown in Figure 5A(a–d). As indicated in the “Curcumin can increase the cytotoxicity of 5-FU on SW480 cells” section, curcumin could significantly enhance the inhibitory effect of 5-FU on the viability of SW480 cells. Therefore, we also measured the ROS level of SW480 cells treated with different concentrations (0, 20, 50, and 100 μM) of 5-FU and 5 μM curcumin. The results showed that ROS levels in the cells treated with both 5-FU and curcumin increased significantly compared to those in the cells treated with 5-FU alone in a dose-dependent manner, as shown in Figures 5A(i–iv),B.

Curcumin is a natural phenolic compound extracted from turmeric. It has been proven that curcumin has multiple biological activities, such as antioxidant, hypotensive, anti-inflammatory, and immune enhancement, especially with high anti-tumor activity. Existing studies have found that curcumin has a significant inhibitory effect on colorectal cancer, thyroid cancer, and liver cancer. Chemotherapy plays a vital role in the comprehensive treatment of tumors, especially in patients with advanced tumors. Curcumin can promote the chemosensitivity of a variety of cancers by multiple mechanisms, including enhancement of the production of intracellular ROS (24–26).

Oxidative stress, which is caused by harmful stimulation, intracellular reactive oxygen species (ROS) level, broken oxidative balance, and excessive ROS, can affect mitochondrial function, inhibit the cell cycle, and, through the mitochondria, cause endoplasmic reticulum stress, the death of receptor regulation pathways, cause DNA damage, and induce cell apoptosis (27). Previous studies have found that curcumin can cause an increase in ROS levels and oxidative stress in CRC cells, thereby inducing cell apoptosis (28). Studies have found that curcumin can significantly increase the ROS level in SGC7901 gastric cancer cells, upregulate the protein expression of Bax and P53, downregulate the protein expression of Bcl-2, and activate the apoptosis mediated by the JNK regulatory pathway (29). The increase in ROS induced by curcumin can also cause ER stress and induce cell apoptosis. Studies have found that curcumin can affect the upregulation of ER stress regulatory protein CHOP and glucose regulatory response protein GRP78 expression in SUNE1 cells of nasopharyngeal carcinoma, activate ER stress, and thus induce cell apoptosis (30).

## Conclusion

The underlying mechanisms of the chemosensitizating activity of curcumin are complicated and multifaceted; the intracellular reactive oxygen species are recognized to play a key role, as revealed by the identification of some oxidized protein targets upon treatment by curcumin in the present study. However, the detailed mechanisms within which these oxidative modifications play a role in the chemosensitazing effect of curcumin have not been well established and need further in-depth investigations *in vitro* and *in vivo*. Overall, our findings in the present study could provide a new direction for further elucidating the sensitization mechanism of curcumin.

## Data availability statement

The raw data supporting the conclusions of this article will be made available by the authors, without undue reservation.

## Author contributions

NL and CH: conceptualization. JY and NL: methodology, writing, review, and editing. All authors have read and agreed to the published version of the manuscript.

## Conflict of interest

The authors declare that the research was conducted in the absence of any commercial or financial relationships that could be construed as a potential conflict of interest.

## Publisher's note

All claims expressed in this article are solely those of the authors and do not necessarily represent those of their affiliated organizations, or those of the publisher, the editors and the reviewers. Any product that may be evaluated in this article, or claim that may be made by its manufacturer, is not guaranteed or endorsed by the publisher.
